# Modeling of SARS-CoV-2 Treatment Effects for Informed Drug Repurposing

**DOI:** 10.3389/fphar.2021.625678

**Published:** 2021-03-10

**Authors:** Charlotte Kern, Verena Schöning, Carlos Chaccour, Felix Hammann

**Affiliations:** ^1^ Clinical Pharmacology and Toxicology, Department of General Internal Medicine, Inselspital (Bern University Hospital), University of Bern, Bern, Switzerland; ^2^ Graduate School for Health Sciences, University of Bern, Bern, Switzerland; ^3^ ISGlobal, Hospital Clínic-Universitat de Barcelona, Barcelona, Spain; ^4^ Clínica Universidad de Navarra, Pamplona, Spain; ^5^ Ifakara Health Institute, Ifakara, Tanzania

**Keywords:** COVID-19, disease modeling, drug repurposing, viral kinetics, pharmacometrics

## Abstract

Several repurposed drugs are currently under investigation in the fight against coronavirus disease 2019 (COVID-19). Candidates are often selected solely by their effective concentrations *in vitro*, an approach that has largely not lived up to expectations in COVID-19. Cell lines used in *in vitro* experiments are not necessarily representative of lung tissue. Yet, even if the proposed mode of action is indeed true, viral dynamics *in vivo*, host response, and concentration-time profiles must also be considered. Here we address the latter issue and describe a model of human SARS-CoV-2 viral kinetics with acquired immune response to investigate the dynamic impact of timing and dosing regimens of hydroxychloroquine, lopinavir/ritonavir, ivermectin, artemisinin, and nitazoxanide. We observed greatest benefits when treatments were given immediately at the time of diagnosis. Even interventions with minor antiviral effect may reduce host exposure if timed correctly. Ivermectin seems to be at least partially effective: given on positivity, peak viral load dropped by 0.3–0.6 log units and exposure by 8.8–22.3%. The other drugs had little to no appreciable effect. Given how well previous clinical trial results for hydroxychloroquine and lopinavir/ritonavir are explained by the models presented here, similar strategies should be considered in future drug candidate prioritization efforts.

## Introduction

Since the beginning of the ongoing global outbreak of severe acute respiratory syndrome coronavirus 2 (SARS-CoV-2), a variety of drug therapies have been proposed. Some are based on expert opinion, some on promising *in vitro* results, some on findings in case series from compassionate or off-label treatments. Unfortunately, whenever they are put through the rigorous process of randomized clinical trials, little evidence for palpable real-world benefits remains.

Novel coronavirus disease 2019 (COVID-19) spreads rapidly not only from host to host but within each host as well. The infection progresses at a staggering speed in individual patients which may become infectious after 2–3 days and reach peak viral loads only a few days after the reverse-transcriptase polymerase chain reaction (RT-PCR) test becomes positive (To et al., 2020). The need for early initiation of drug therapy has been recognized as key for successful treatment of infectious diseases, and COVID-19 is unlikely to be an exception (Gonçalves et al., 2020).

The repurposing of drugs with established supply chains and low manufacturing costs seems the straightest path towards a timely pharmacological intervention. Because our understanding of the pathophysiology of COVID-19 is still evolving, the selection of viable candidates is mostly dictated by extrapolations from *in vitro* and *in silico* evidence. Identified drug targets include the viral structural spike (S) protein; the host type 2 transmembrane serine protease (TMPRSS2); 3-chymotrypsin-like (3CL) protease mediating proteolysis; RNA-dependent RNA polymerase; and interleukin-6 receptors (Arshad et al., 2020; Sanders et al., 2020).

A basic tenant of clinical pharmacology states that an unbound drug must reach its target at sufficient concentrations (e.g., half-maximal effective target concentrations (EC_50_)) and maintain them to exert effects. This is a common criterion for drug candidate selection and has been applied to COVID-19 early on in well-conducted comprehensive surveys (Arshad et al., 2020). Unfortunately, the candidates with highest probability of success have largely failed in practice, and it appears that the EC_50_ approach might be too simplistic for this disease, as it is important not only whether EC_50_ is reached, but also for how long concentrations (above EC_50_) can be maintained, especially at the target site.

One reason may be the failure to account for host factors. For instance, a crucial element of treatment response is host immunity. There are *in vivo* studies on the temporal dynamics of immune response and seroconversion (Long et al., 2020; To et al., 2020; Young et al., 2020). Early suppression of viral load even for brief periods may be beneficial by providing more time for the host to mount a defense and assist in clearing an otherwise overwhelming infection.

The viral kinetics of several diseases have been successfully described mathematically in the past, e.g., influenza, hepatitis C, or Ebola (Beauchemin and Handel, 2011; Canini and Perelson, 2014; Oakes et al., 2018). For COVID-19, Kim et al. used a target cell limited model to show the importance of early initiation of treatment and drug mode of action (Kim et al., 2020b). Other authors arrive at similar results with eclipse models (Czuppon et al., 2020; Gonçalves et al., 2020; Hernandez-Vargas and Velasco-Hernandez, 2020). None of these studies, however, directly used pharmacokinetic profiles in their models.

With this modeling and simulation study, we aimed to understand the influence of different modes of action, concentration profiles, dosing schedules, and timing of interventions on key parameters of viral load (peak load, duration of positivity, and total exposure as measured by area under the curve (AUC)) in acute COVID-19. We developed a model of the within-host viral kinetics of SARS-CoV-2 from published patient data and drove antiviral effect with simulated pharmacokinetic (PK) profiles of selected drugs with different dose regimens. These drugs include hydroxychloroquine (HCQ, considered a blocker of viral entry), ritonavir-boosted lopinavir (LPV/r, a 3CL inhibitor), ivermectin (IVM, a broad spectrum anthelminthic with antiviral activity), nitazoxanide (NZT, an antiparasitic agent with antiviral activity), and artemisinin (ART, the primary component of sweet wormwood, believed to inhibit viral entry and intracellular reproduction of SARS-CoV-2) (Caly et al., 2020; Choy et al., 2020; Gordon et al., 2020; Li et al., 2005; Liu et al., 2020; Wang et al., 2020a; Wu et al., 2020). Our selection was influenced by perceived research interest (HCQ, LPV/r, NZT) and lay use of drugs in the general public as self-medication (HCQ, IVM, ART) (Martins-Filho et al., 2020; Molento, 2020; Nordling, 2020; Owens, 2020; WHO, 2020). Although remdesivir has so far shown the greatest promise, there is currently not enough published data to allow for pharmacometric simulation in the model proposed here, and hence the drug was not included (Beigel et al., 2020).

## Methods

### Data Sources

Viral kinetic profiles of COVID-19 patients were taken from Young et al. (2020), a study that followed the first patients (*n* = 18) in four hospitals in Singapore (Chinese nationals: *n* = 16, Singapore residents: *n* = 2). We read out values using a digitizing software. Most (*n* = 13) were not on specific therapy and were included in the analysis. Viral load was measured from nasopharyngeal swabs with RT-PCR and presented in cycle threshold (Ct) values (Young et al., 2020). As the correlation between Ct values and viral load varies by laboratory and analytical conditions, we chose to relate model output with observed Ct values with a published regression fit (Chu et al., 2020). Since the time of infection was not recorded, this value had to be estimated. Although the incubation period varies between patients, an average incubation period of 5 days fitted well for all patients (Lauer et al., 2020). We fixed the positivity threshold at 35 cycles, corresponding to 10^1.58^ copies/ml (Wang et al., 2020b).

### Viral Kinetics Models

In the standard target cell limited model, virus particles *V* infect a pool of susceptible (target) cells *T* with the cellular infection rate *β*. Infected cells *I* begin shedding virions at a production rate *p* (Canini and Perelson, 2014). The parameters *c* and *δ* determine the rate of clearance of virus and cell death of infected cells, respectively. The time-dependent number of susceptible cells (Eq. (1)), infected cells (Eq. (2)), and viral load (Eq. (3)) are described by a system of ordinary differential equations as follows:

$$\frac{dT}{dt}=\;-\left(1-\eta \right)\beta TV,$$ (1)

$$\frac{dI}{dt}=\left(1-\eta \right)\beta TV-\;\delta I,$$ (2)

$$\frac{dV}{dt}=\left(1-\varepsilon \right)pI-cV.$$ (3)

The effects of pharmacological treatments by different modes of action are described by the following variables: inhibition of viral entry into susceptible cells, by decreasing the cellular infection rate with effectiveness η, and/or by blocking viral production rate within infected cells with effectiveness ε. We modeled treatment effect based on the IC_50_ or EC_50_ values of the drugs on their respective targets using a sigmoidal E_max_ model (Eq. (4)), with *C(t)* being the concentration of the drug at a given time:

$$\varepsilon \;or\;\eta =\;\frac{E_{max}\;\times C\left(t\right)}{EC_{50}+C\left(t\right)}.$$ (4)

We also considered an eclipse model, an extension in which infected cells enter an eclipse phase (*E*) for an average duration $k^{-1}$ until they begin shedding virions. Initial conditions were set as

$$T\left(0\right)=T_{0},$$

$$V\left(0\right)=V_{0},$$

I(0)=0,

and additionally, for the eclipse model,

E(0)=0,

where T_0_ is the number of susceptible cells fixed to $1\times 10^{5}$ (based on prior modeling efforts and accounting for ∼1% of alveolar cells expressing ACE2, the main point of entry for SARS-CoV-2) (Baccam et al., 2006; Li et al., 2020b), V_0_ the initial viral load on inoculation (fixed at $1\times 10^{0}$ copies/ml), and E_0_ the number of cells in eclipse state. The within-host reproduction number R_0_ was set to 3.79 (Li et al., 2020a). This value is also approximately in the same range as other within-host virus kinetic models (Kim et al., 2020b; Hernandez-Vargas and Velasco-Hernandez, 2020). Other parameters need to be estimated by numerical optimization, i.e., viral clearance *c*, the production rate *p*, and the death rate of infected cells *δ*. The cellular infection rate *β* of the virus is dynamically calculated:

$$\beta =\frac{c\delta R_{0}}{\left(p-\delta R_{0}\right)T_{0}}.$$ (5)

Supplementary Table S2 shows all model parameters and sources.

### Immune Response

Our understanding of SARS-CoV-2 immunity is still evolving. Immunity could involve cells entering into a refractory state or an antibody-mediated increase in viral clearance. Adding an additional state would increase model complexity beyond what seems supported by the source data. We therefore chose to enter acquired immune response as a time-dependent covariate effect on viral clearance *c*. Temporal dynamics are based on Long et al. (2020) who evaluated seroconversion for IgM and IgG in 285 patients from three hospitals in Chongqing (neighboring Hubei Province). Data were extracted with a digitizing software and fitted to a sigmoidal E_max_ model. As the effect size of the immune response in SARS-CoV-2 infection (E_max, immunity_) is unknown, we estimated this value together with the models of viral kinetics.

### Pharmacokinetic Models

We simulated pharmacokinetics (PK) of HCQ, IVM, LPV/r, and ART from published population pharmacokinetics models. Profiles for HCQ were simulated from healthy volunteers reported by Lim et al. (2009). The IVM model was taken from Duthaler et al. and simulated using fed state dosing (Duthaler et al., 2019). The LPV/r model by Dickinson et al. (2011) was built from data of healthy volunteers receiving 400/100 mg, the dose that was under investigation in WHO Solidarity. For ART we directly implemented the model developed by Birgersson et al. in healthy male volunteers with a dosing regimen of 500 mg daily for 5 days (similar to historical dosing recommendations in malaria) (Birgersson et al., 2016). No published pharmacometric model is available for NTZ. The drug is rapidly and completely hydrolysed to an active metabolite, tizoxanide (TZ). We therefore extracted the mean TZ pharmacokinetic profile from a study in healthy Mexican volunteers with a digitizing software, fitted a one-compartment oral absorption model with lag time, and used this for simulation (Balderas-Acata et al., 2011).

As the protein-bound fraction of a drug is considered not interacting with its target, we only considered the unbound fractions of the drugs to be available (Supplementary Table S2), i.e. 50% for HCQ (Furst, 1996), 7% for IVM (Klotz et al., 1990), 1% for NTZ (FDA, 2005), and 1% for LPV (Boffito et al., 2004). No human *in vivo* data exist for lung concentrations in any of the drugs in this study. We used literature-based approximations to adjust for differences between plasma and lung concentration profiles. The issue of lung tissue concentrations is particularly contentious for HCQ, with some reports of lung:plasma ratio ranging from 27 to 177 in macaques (Maisonnasse et al., 2020). Recent evidence suggests that in COVID-19 HCQ plasma concentrations are more representative (Fan et al., 2020). For IVM lung accumulation, we used cattle data published by Lifschitz et al., an approach also used in another publication discussing the potential role of IVM in COVID-19 (Lifschitz et al., 2000; Schmith et al., 2020). LPV concentrations in lung tissue were assumed to be 1.78 times higher than in plasma, and protein binding was set to 99% (Atzori et al., 2003; FDA, 2013). For NTZ we used estimates from a recently prepublished physiology-based pharmacokinetic (PBPK) model for lung partitioning (Rajoli et al., 2020).

### Pharmacodynamic Effects

The effectiveness of HCQ was shown *in vitro* in Vero E6 cells by Liu et al. (2020). The EC_50_ values at 48 h ranged between 4.06 and 12.96 µM, depending on the amount inoculated. We entered the mean of these values (8.51 µM) as an effect on the reduction of the cellular infection rate *β*. We simulated dosages of 200 mg q8h for 10 days as proposed by Gautret et al. and the scheme previously employed in the WHO Solidarity trial, 800 mg q12 h on the first day (loading dose) and 400 mg q12 h on days 2–10 (Gautret et al., 2020; WHO, 2020).

For IVM, we assumed two pharmacodynamic effects: the inhibition of RNA helicase and inhibition of nicotinic acetylcholine receptors (nAChR). The inhibitory effect of IVM on helicase has been previously reported for flaviviridae, i.e., yellow fever virus (YFV, IC_50_ 0.12 µM), Dengue virus (DENV, IC_50_ 0.5 µM), and West Nile Virus (WNV, IC_50_ 0.35 µM) (Mastrangelo et al., 2012). There are no *in vitro* data for SARS-CoV-2 yet, although Caly et al. (2020) have reported a strong maximal inhibition of virus replication in the Vero E6 cell line with an IC_50_ of about 2 µM. Higher concentrations (10–25 µM) need to be achieved for similar inhibition of DENV replication (Wagstaff et al., 2012). The difficulties in achieving micromolar concentrations have led some authors to speculate IVM is not druggable in the context of COVID-19 (Bray et al., 2020). Strikingly, despite the higher IC_50_ in DENV infected Vero E6 cells, a small trial of IVM 3 × 400 µg/kg in DENV patients demonstrated antiviral effects *in vivo* (Yamasmith et al., 2018). Due to the higher susceptibility of SARS-CoV-2 to IVM than DENV in Vero E6 cells, we used a conservatively reduced IC_50_ of 0.1 µM in the simulations as an inhibitory influence on viral production *p*.

In addition, IVM interacts with nAChR (IC_50_ 156 nM) (Degani-Katzav et al., 2017). It has been hypothesized that inhibition of nAChR downregulates angiotensin-converting enzyme 2 (ACE2) expression and thus reduces the points of entry for SARS-CoV-2 (Oakes et al., 2018). We enter this as a net inhibitory effect on the cellular infection rate *β*. In contrast to direct inhibition of viral entry, this is an antiviral activity mediated by the host and therefore not easily captured in *in vitro* assays. For IVM, we evaluated 300 µg/kg and 600 µg/kg q24 h for 3 days. These dosages are not approved, but safety and tolerability of single fixed doses of 120 mg were shown previously in healthy volunteers (Guzzo et al., 2002).

LPV and RTV are both protease inhibitors. Their use in COVID-19 was investigated as a now discontinued arm of the WHO solidarity trial (LPV 400 mg and RTV 100 mg q12 h for 14 days) (WHO, 2020). LPV reduced the viral RNA copies of SARS-CoV-2 *in vitro* with an EC_50_ of 26.1 µM, whereas RTV has an EC_50_>100 µM (Choy et al., 2020). As RTV in this coformulation (LPV/r) is only intended to boost the bioavailability of LPV, we only consider the antiviral effect of LPV on the viral production rate *p* (Chandwani and Shuter, 2008).

ART as the main component of *A. annua* (sweet wormwood) extract has not been studied in SARS-CoV-2. Nair et al. reported an antiviral effect in Vero E6 cells of artesiminin on SARS-CoV-2 with an EC_50_ of 19.8 µg/ml (=70 µM) (Nair et al., 2021). Studies suggest that artemisinin interferes with viral entry by interaction with the spike protein (Sehailia and Chemat, 2020), but it also affects postentry steps of infection (Cao et al., 2020b; Nair et al., 2021). We entered this as an effect on viral production rate *p* and the cellular infection rate *β*.

NTZ has shown *in vitro* activity against SARS-CoV-2 in Vero E6 cells at an EC_50_ of 2.12 µM (Wang et al., 2020a). The mechanism of action is unclear, but it has been hypothesized that NTZ inhibits viral entry as well as replication. We used both effects in the simulations (Arshad et al., 2020).

### Software

We modelled and simulated pharmacokinetic profiles with Pkanalix and mlxR (version 4.1.3), an R package for interfacing with Monolix (version 2019R2, http://www.lixoft.com, Antony, France). Data for viral loads and NTZ were read out with WebPlotDigitizer (version 4.2, https://automeris.io/WebPlotDigitizer). Immunity E_max_ and EC_50_ were estimated using the R package rstanemax (version 0.1.2). Data checkout, analysis, and visualization were performed in GNU R (version 3.6.3, R Foundation for Statistical Computing, http://www.R-project.org, Vienna, Austria). Ordinary differential equation (ODE) systems and parameter estimations were implemented with the R packages deSolve (version 1.28) and dfoptim (version 2018.2-1).

## Results

### Viral Kinetics Models

We used the viral load profiles of untreated patients published by Young et al. (*n* = 13, Supplemental Material) (Young et al., 2020). We evaluated target cell limited and eclipse models, both with a time-varying effect on viral clearance *c* following a sigmoidal E_max_ model fitted to reported seroconversion data (Long et al., 2020). The averaged parameters estimates from individual profiles with the Nelder-Mead method were as follows (see also Supplementary Table S1 and Supplementary Figure S1):

- viral clearance *c:* 5.07,
- production rate *p:* 10.2,
- death rate of infected cells *δ*: 0.54, and
- maximal immune effect on clearance E_max,immunity_: 57.0.

Nonlinear mixed effects implementations of these models proved less robust to changes in initial estimates and suffered from numerical identifiability problems.

Profiles were best described by a standard target cell limited model. The addition of an eclipse phase did not improve fits and also introduced identifiability issues, as was already noted in another study (Hernandez-Vargas and Velasco-Hernandez, 2020). Left untreated, viral load exceeds the RT-PCR positivity threshold of 35 cycles at 5.4 dpi, peaks at 10.2 dpi with a Ct value of 28.4 cycles, and drops below the positivity limit at 18.9 dpi, similar to reports from clinical studies (Kim et al., 2020a; Lauer et al., 2020; To et al., 2020). Total viral exposure (measured as AUC) was 12’003 days*log(copies/ml).

### Dosage and Effectiveness of Treatment

**FIGURE 1:**
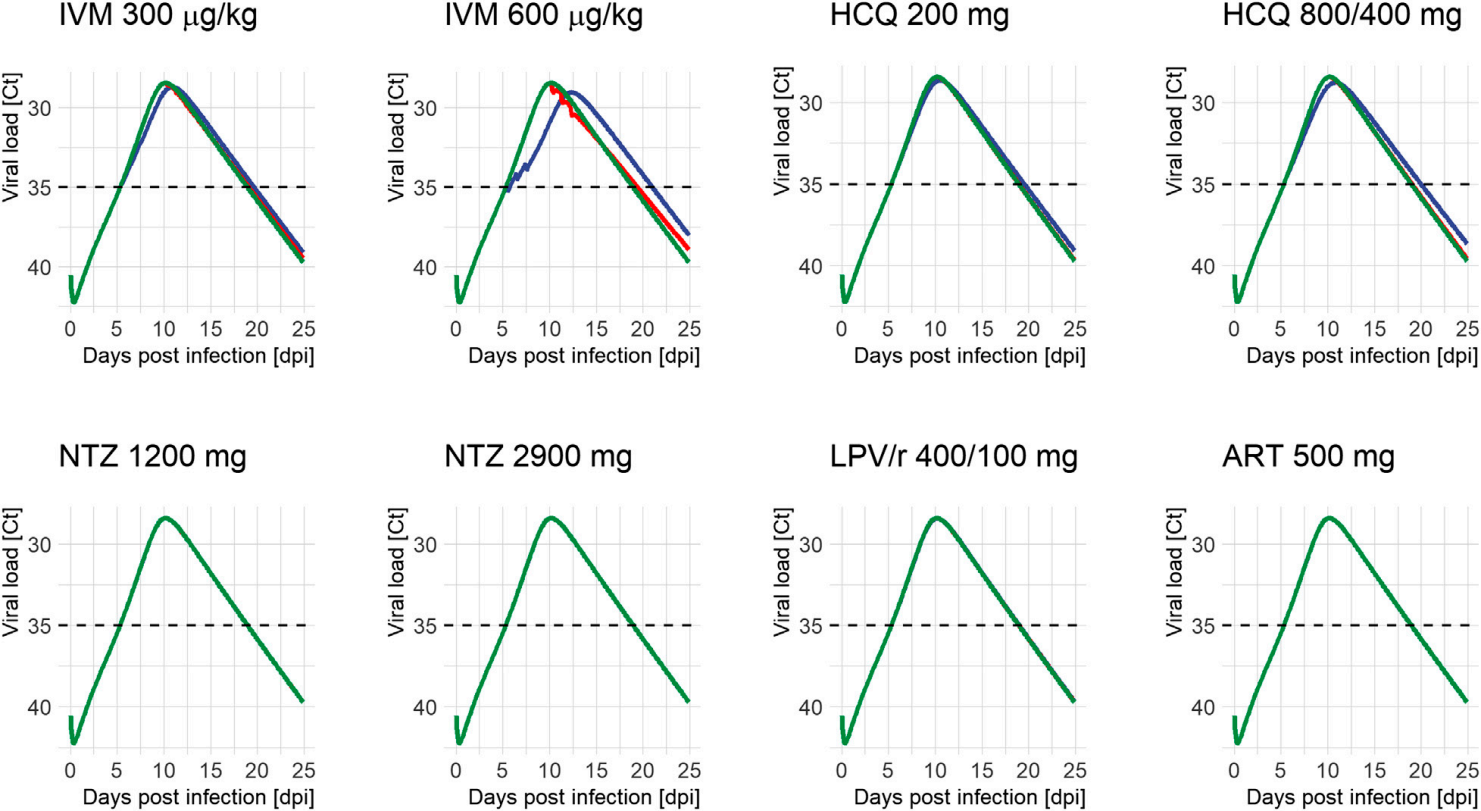
Viral load profiles of SARS-CoV-2 following different treatment regimens and initiation of treatment (green: untreated, blue: on positivity (5.4 days after infection), and red: on peak (10.2 days after infection)). Lines may overlap so that only one color is visible; simulations were always run for all time points. Ct: serial cycle threshold values; ART: artemisinin; HCQ: hydroxychloroquine; IVM: ivermectin; LPV/r: lopinavir/ritonavir; NTZ: nitazoxanide. Dosing of different modeled treatment regimens: IVM 300: 300 μg/kg every 24 h for 3 days; IVM 600: IVM 600 μg/kg every day for 3 days; HCQ 200: 200 mg every 8 h for 10 days; HCQ 800/400: 800 mg every 12 h for 1 day, then 400 mg every 12 h for 9 days; NTZ 1200: NTZ 1200 mg every 6 h for 5 days; NTZ 2900: NTZ 2900 mg every 12 h for 5 days; LPV/r 400/100: LPV/r 400/100 mg every 12 h for 14 days; ART 500: ART 500 mg once a day for 5 days.

**FIGURE 2:**
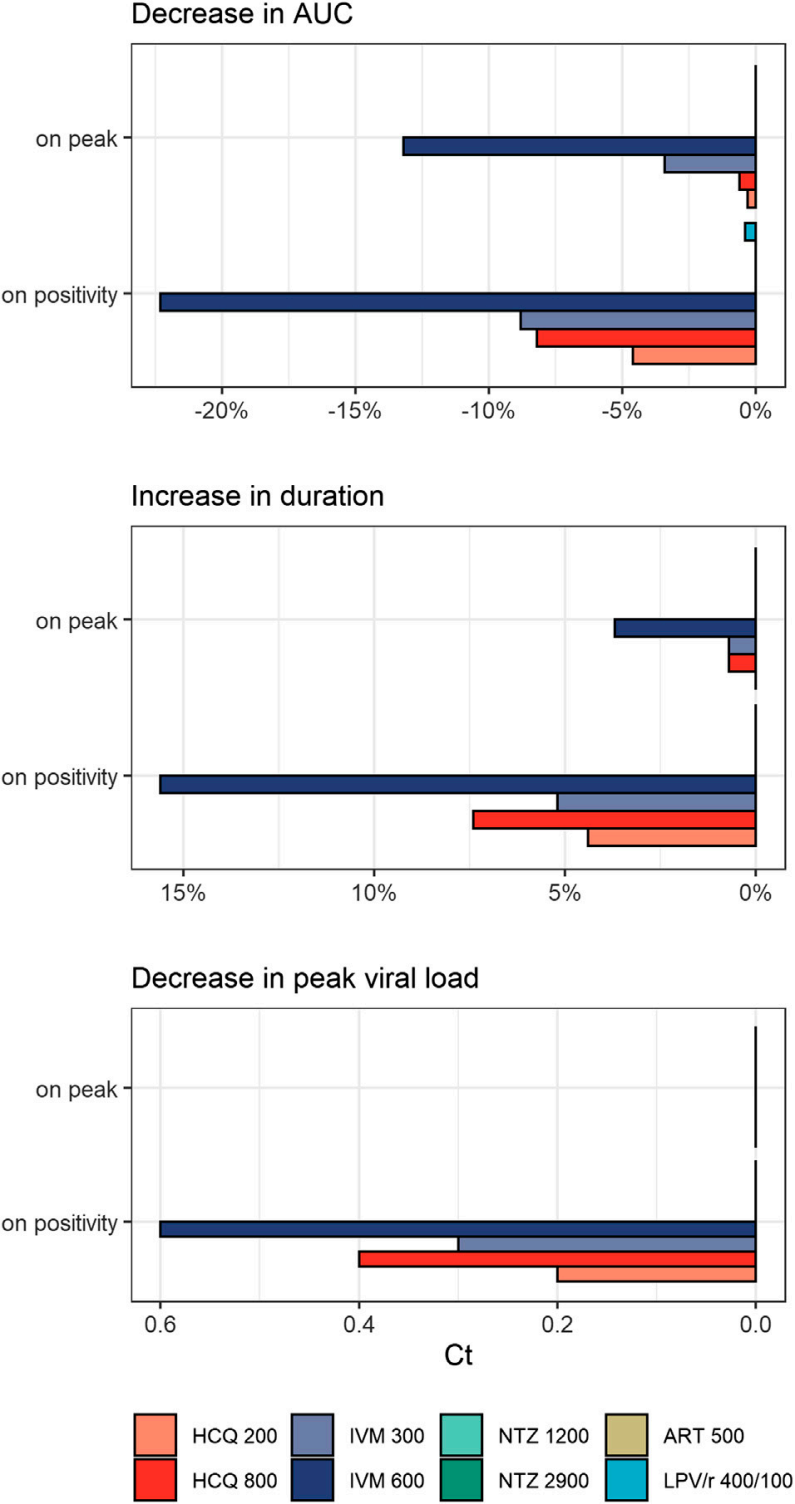
Treatment effects on viral exposure as difference in area under the curve (AUC), relative change in duration, and change in peak cycles (Ct) following different times of treatment initiation (on positivity: 5.4 days after infection, on peak: 10.2 days after infection). HCQ: hydroxychloroquine; IVM: ivermectin; NTZ: nitazoxanide; ART: artemisinin; LPV/r: lopinavir/ritonavir. Dosing of different modeled treatment regimens: HCQ 200: 200 mg every 8 h for 10 days; HCQ 800: 800 mg every 12 h for 1 day, then 400 mg every 12 h for 9 days; IVM 300: 300 μg/kg every 24 h for 3 days; IVM 600: IVM 600 μg/kg every day for 3 days; NTZ 1200: NTZ 1200 mg every 6 h for 5 days; NTZ 2900: NTZ 2900 mg every 12 h for 5 days; ART 500: ART 500 mg once a day for 5 days; LPV/r 400/100: LPV/r 400/100 mg every 12 h for 14 days.

Temporal impact of treatment is shown as individual curves in Figure 1. Effect on viral exposure as difference in area under the curve (AUC), relative change in duration, and change in peak cycle (Ct) are presented in Figure 2. Full results including changes in peak viral load and duration of disease are available in Supplementary Table S3 and Supplementary Figure S2. The PK curves of the treatments and the corresponding effect on SARS-CoV-2 viral kinetics are shown in Supplementary Figures S2–S5.

HCQ reduced peak viral load by 0.2–0.3 log units and exposure by 4.6–8.2% when given on positivity. Treatment starting around peak viral load (10.2 dpi) had no appreciable effect on total viral load or duration of disease. Between both dose regimens, the WHO Solidarity trial arm resulted in the more pronounced reduction in total viral load. Effects of IVM were even more pronounced: given on positivity, peak viral load dropped by 0.3–0.6 log units and exposure by 8.8–22.3%. Exposure reductions are associated with slightly prolonged durations of shedding from 13.5 days (untreated) to 14.2–15.6 days for IVM and 14.1–14.5 days for HCQ, and a shift of T_max_ from day 10.2 (untreated) to day 10.9–12.3 and day 10.5–10.9, respectively. Interestingly—and in contrast to HCQ—some effects remain when treatment is initiated around peak viral load (3.4-13.2% difference in exposure). LPV/r, ART, and NTZ had no influence on viral dynamics, independent of time of initiation.

## Discussion

Our modeling and simulation study described patient viral load well and captured the essential milestones of SARS-CoV-2 viral kinetics, e.g., duration of viral shedding and peak viral loads. It also shows that the window of opportunity to treat COVID-19 is narrow. As the infection spreads rapidly throughout the host, the pool of susceptible cells is quickly depleted. Drugs inhibiting viral entry (like HCQ) therefore only appear to have a role, if any, in the first days after inoculation (post-exposure prophylaxis) or as primary prophylactic agents handed out to at-risk individuals.

These findings may help to explain the disappointing results of clinical trials with HCQ: by the time patients are hospitalized or even transferred to critical care, few susceptible cells are left, so little impact can be made at this point (Annie et al., 2020; Cavalcanti et al., 2020; Molina et al., 2020; Tang et al., 2020a). The WHO Solidarity trial’s dosing scheme was clearly more effective than the one proposed by Gautret et al. (2020). However, even with the higher dosing scheme used in the WHO Solidarity trial, no appreciable effect of HCQ was observed and the treatment arm was prematurely terminated on June 18, 2020 (Pan et al., 2020). Of note, recent trials have also failed to find benefits for HCQ in pre- and post-exposure prophylactic indications (Boulware et al., 2020; Rajasingham et al., 2020). Since viral load is not the only determinant of disease state, one cannot directly deduce clinical effect of any of the regimens from these simulations. Given the negative results of previous trials with HCQ, we suggest that HCQ results should be used as a lower threshold to rank other drugs against.

We found greatest effects for IVM. Again, the earlier and longer the exposure, the better, but compounds like IVM still convey some benefit if initiated at a later stage. When held to the HCQ benchmark, IVM 600 µg/kg daily for 3 days, particularly when given around time of positivity, may have meaningful impact whereas IVM 300 µg/kg daily for 3 days had efficacy comparable to HCQ regimens. This finding is in contrast to other analyses suggesting IVM is poorly druggable in COVID-19 (Schmith et al., 2020). It is important to stress that these IVM doses, while apparently safe in healthy volunteers, are far higher than any dose approved for other indications (1 × 200 µg/kg to 1 × 400 µg/kg). At 3 × 600 µg/kg in a 70 kg patient, doses are similar to the maximum doses (120 mg single administration) described by Guzzo et al. (2002). Boosting exposure to IVM by co-administering inhibitors of its metabolism or elimination (such as the CYP3A4 and P-glycoprotein (P-gp) inhibitor ritonavir) is a theoretical option (Chaccour et al., 2017). However, there are concerns that inhibition of P-gp as an integral part of the functional blood brain barrier could lead to more central nervous adverse events (Chandler, 2018). Until this interaction has been studied systematically, it seems unwise to explore this strategy. For IVM, no results of clinical trials regarding its effectiveness in COVID-19 have been published yet.

ART, NTZ, and LPV/r had no noteworthy effect and do not appear suitable candidates for follow-up at this point. We attribute this in part to their strong protein binding (88–99%), leaving little free drug to engage with targets. Additionally, the EC_50_ for ART is rather high at 70 µM and not likely to be even partially achieved. For LPV/r our findings are confirmed by clinical trial results (Cao et al., 2020a, 24), notably the RECOVERY trial (University of Oxford, 2020) and the WHO Solidarity trial, which discontinued the LPV/r treatment arm on July 4, 2020 (Pan et al., 2020). As of now, no trials on NTZ and ART have reported results.

Our study has several limitations. Our model parameter estimates are based on assumptions of incubation time and number of target cells in the lungs, both of which introduce bias. COVID-19 was initially described from a cluster of pneumonia cases, and while symptoms of the upper and lower airways are most recognizable, vascular, thromboembolic, gastrointestinal, and neurological symptoms have been widely described (Klok et al., 2020; Mao et al., 2020; Struyf et al., 2020; Zhang et al., 2020). Expression levels of ACE2 are also much higher in other tissues, e.g., the small intestine, the kidneys, and the heart (Li et al., 2020b). Hence it seems unlikely that systemic viral loads are solely a product of alveolar epithelial cells. Extent of viral burden is dependent on disease severity and also site of sampling (e.g., oropharyngeal, nasopharyngeal, and plasma (Fajnzylber et al., 2020)). Ethnicity is thought to affect clinical outcomes (Sze et al., 2020), yet there is inconclusive evidence as to the impact of ethnicity on viral load (Magleby et al., 2020). In conclusion, we suggest that the size of the pool of target cells be reestimated in different populations.

Point estimates of viral kinetics parameters yielded realistic estimates of viral load profiles with reasonable uncertainty around point estimates (%CV: 30–43). We did not normalize the asynchronous dynamics of the source data (e.g., viral peak at different dpi), which might have improved fits. However, currently no accepted procedure exists (Hernandez-Vargas, 2019). Other authors have used more sophisticated methods such as nonlinear mixed effects (nlme) modeling on the same source data, implementing other structural models such as eclipse models (Czuppon et al., 2020; Gonçalves et al., 2020). Our nlme implementations of the models suffered from the same numerical identifiability issues seen by other authors (Hernandez-Vargas and Velasco-Hernandez, 2020). Given that the model presented here is structurally different from other target cell limited or eclipse models, some point estimates can differ from other implementations (such as the reproduction number R_0_, or number of susceptible cells T_0_ (Gonçalves et al., 2020)).

Virus extinction is not captured by any models like the one proposed here. We therefore decided not to model prophylactic dosing (prior to or on exposure) and resorted to return to negativity on RT-PCR as a surrogate measure for disease duration. This is supported by data suggesting that late stage shedding is of noninfectious virus particles only (Walsh et al., 2020).

We made several assumptions on modes of drug action and their efficacy. We used the published mode of action of HCQ on SARS-CoV-2, which were performed in the African green monkey kidney-derived cell line Vero (Wang et al., 2020a), even though there is evidence that this cell line might not be suitable to represent lung tissue (Hoffmann et al., 2020). The model places HCQ effects only on viral entry, although it might also have other modes of actions which might affect the production of virions within infected cells (Quiros Roldan et al., 2020; Tripathy et al., 2020). Additionally, HCQ has immunomodulatory effects and might hinder the activation of B and T cells and thus inhibit the host-innate immune response (Goldman et al., 2000; Quiros Roldan et al., 2020). No data for SARS-CoV-2 were available for modeling. If relevant at all, our model would be overestimating the effectiveness of HCQ.

The *in vitro* evidence of efficacy of IVM against SARS-CoV-2 is not detailed enough to model effects with greater precision. Based on data from other flaviviridae, particularly Dengue virus, the proposed inhibition of replication seems reasonable (Mastrangelo et al., 2012). Inclusion of effects on viral entry follows pathophysiological reasoning and has yet to be confirmed in studies. IVM engages with nAChR, which leads to a reduction of ACE2 and in turn decreases the points of entry for SARS-CoV-2, influencing the cellular infection rate. Even though the IC_50_ for IVM on nAChR has been experimentally determined, we do not know how exactly this relates to the reduction of ACE2 expression. Therefore, we decided to model it solely based on plasma levels and the available IC_50_.

In HCQ and IVM, even though total viral load is reduced, the duration of virus shedding might be increased, a consequence of a ‘flattening of the curve’ similar to what is observed on a population scale. However, this gives the immune response more time to develop an immune response. For this reason, overall total viral load decreases in the simulated models. Changes in peak viral load were moderate at best (<1 log unit). While being convenient endpoints to measure in a clinical setting, AUCs of viral load appear more appropriate for drug discovery.

ART and NZT have only recently received attention in COVID-19 treatment. Despite having little effect in our study, they would make excellent candidates from economic and logistic points of view. We selected ART as it is the primary active ingredient in sweet wormwood. Herbal concoctions of wormwood are being promoted as a cheap, easily accessible form of self-medication in COVID-19 (Welle, 2020). *A. annua* and derivatives (artesunate, dihydroartesunate) are widely used as antimalarials. Effective concentrations have yet to be determined for ART and its derivatives, although the large degrees of protein binding imply that effective target concentrations need to be in the low micromolar range. Studies suggest that artemisinin has also anti-inflammatory and immunomodulatory effects, which might be beneficial when treating COVID-19 (Tang et al., 2020b). However, as these effects are not related to viral kinetics, we were not able to include these in our simulations. Even then its use would have one crucial limitation: the WHO is discouraging use of oral artemisinin monotherapy (AMT) in malaria as it is considered to be a major factor for the development of parasite resistance (WHO, 2014). A renaissance of oral AMT in malaria endemic regions during the ongoing COVID-19 pandemic could cause more harm than good.

For NTZ, pharmacokinetic simulations were based on a mean curve of a single dose of NTZ 500 mg. The EC_50_ value was determined for NTZ, not the immediately formed active metabolite tiaxozanide, which would be the active compound expected to reach tissue (Wang et al., 2020a). When more detailed results for tiaxozanide are available, simulated efficacy could change.

In conclusion, while *in vitro* studies are very well suited to identify possible modes of actions of potential treatments for COVID-19, they are unable to predict the clinical efficacy of a drug. Our simulation of treatments fitted well with available results from clinical trials, even though several estimations had to be made and limitations accepted. Although early initiation was a strong determinant for treatment effect, none of the interventions studied showed major impact on viral dynamics. Efforts should focus on identification of more efficacious drug candidates and vaccine development. Until then, general social and hygiene measures remain the best interventions to combat COVID-19.

## Data Availability

The original contributions presented in the study are included in the article/Supplementary Material, further inquiries can be directed to the corresponding author. The source code is available on GitHub: https://github.com/cptbern/sars2-viral-kinetics.
