# Supplementary material for: Modeling of SARS-CoV-2 Treatment Effects for Informed Drug Repurposing
Source: Front Pharmacol. 2021 Mar 10;12:625678. doi: 10.3389/fphar.2021.625678 (PMC7988345; doi:10.3389/fphar.2021.625678)
Supplement: Supplementary file 1 [file datasheet1.docx]

Supplementary Material

# Supplementary Tables

**Supplementary** **Table S1** – Summary statistics of individual fits of viral load data. c: viral clearance, δ: infected cell clearance, p: viral production rate, E_max,immunity_: maximum acquired immunity effect on viral clearance, SD: standard deviation, %CV: coefficient of variation (100*mean/SD).

|  | **c** | ***δ*** | **p** | **E_max,immunity_** |
| --- | --- | --- | --- | --- |
| **mean** | 5.07 | 0.54 | 10.2 | 57.0 |
| **SD** | 2.18 | 0.16 | 4.3 | 23.4 |
| **%CV** | 43% | 30% | 42% | 41% |

**Supplementary** **Table S2** - Viral kinetics model parameters. EC_50_: half maximal effective concentration; IC_50_: half maximal inhibitory concentration; HCQ: hydroxychloroquine; IVM: ivermectin; NTZ: nitazoxanide; ART: artemisinin; LPV: lopinavir

| **Parameter** | **Definition** | **Value** | **Reference** |
| --- | --- | --- | --- |
| *β* | Cellular infection rate | $\beta=\frac{R_{0}c\delta}{T_{0}(p-R_{0}\delta)}$ | Calculated |
| *δ* | Infected cell death rate | 0.54 | Estimated |
| *p* | Viral production rate | 10.2 | Estimated |
| *c* | Viral clearance | 5.07 | Estimated |
| *R_0_* | Within-host reproduction number | 3.79 | (Li et al., 2020) |
| *T_0_* | Initial target cells | 10^5^ | Fixed by authors |
| *V_0_* | Initial virus load (inoculum) | 10^0^ | Fixed by authors |
| *EC_50, immunity_* | EC_50_ for immune response | 10.2 | Estimated from (Long et al., 2020) |
| *Hill _immunity_* | Slope of dose-response curve | 3.4 | Estimated from (Long et al., 2020) |
| *E_max_, _immunity_* | Maximum effect on viral clearance | 57.0 | Estimated |
| HCQ | EC_50_ _viral entry_ | 8.51 µM | (Liu et al., 2020) (averaged) |
|  | Protein binding | 50% | (Furst, 1996) |
| IVM | IC_50 helicase_ | 0.1 µM | Fixed by authors |
|  | IC_50 nAChR_ | 0.156 µM | (Degani-Katzav et al., 2017) |
|  | Protein binding | 93% | (Klotz et al., 1990) |
|  | Lung accumulation | 2.6 | (Lifschitz et al., 2000) |
| NTZ | EC_50_ | 2.12 µM | (Wang et al., 2020) |
|  | Protein binding | 99% | (FDA, 2005) |
|  | Lung accumulation | 0.7 | (Rajoli et al., 2020) |
| ART | EC_50_ | 70 µM | (Nair et al., 2021) |
|  | Protein binding | 88% | (Jagdev S. Sidhu, 1997) |
| LPV | IC_50_ _protease_ | 26.63 µM | (Choy et al., 2020) |
|  | Protein binding | 99% | (Boffito et al., 2004) |
|  | Lung accumulation | 1.78 | (Atzori et al., 2003) |

**Supplementary Table S3** - Summary results of viral load simulations. dpi: days post infection; d: day; min Ct: minimum serial cycle threshold values; tmax: time to peak concentration; ΔAUC%: percentage difference in area under the curve; HCQ: hydroxychloroquine; ART: artemisinin; IVM: ivermectin; LPV/r: lopinavir/ritonavir; NTZ: nitazoxanide. Treatments were initiated either on positivity (5.4 dpi) or on peak (10.2 dpi). Dosing of different modeled treatment regimens: HCQ 200 mg every 8h for 10 days; HCQ 800 mg every 12h for 1 day, then 400 mg every 12h for 9 days; IVM 300 µg/kg every 24h for 3 days; IVM 600 µg/kg every day for 3 days; NTZ 1200 mg every 6h for 5 days; NTZ 2900 mg every 12h for 5 days; ART 500 mg once a day for 5 days; LPV/r 400/100 mg every 12h for 14 days.

| **Treatment** | **Timing** | **Dosage** | **Start positivity** | **Duration** | **Ct_min_** | **T_max_** | **ΔAUC%** |
| --- | --- | --- | --- | --- | --- | --- | --- |
| ART 500 | on positivity | 500 mg qd 5d | 5.4 | 13.5 | 28.4 | 10.2 | 0.0 |
| ART 500 | on peak | 500 mg qd 5d | 5.4 | 13.5 | 28.4 | 10.2 | 0.0 |
| HCQ 200 | on positivity | 200 mg q8h 10d | 5.4 | 14.1 | 28.6 | 10.5 | -4.6 |
| HCQ 200 | on peak | 200 mg q8h 10d | 5.4 | 13.5 | 28.4 | 10.2 | -0.3 |
| HCQ 800 | on positivity | 800 mg q12h 1d,  400 mg q12h 9d | 5.4 | 14.5 | 28.8 | 10.9 | -8.2 |
| HCQ 800 | on peak | 800 mg q12h 1d,  400 mg q12h 9d | 5.4 | 13.6 | 28.4 | 10.2 | -0.6 |
| IVM 300 | on positivity | 300 µg/kg q24h 3d | 5.4 | 14.2 | 28.7 | 10.9 | -8.8 |
| IVM 300 | on peak | 300 µg/kg q24h 3d | 5.4 | 13.6 | 28.4 | 10.2 | -3.4 |
| IVM 600 | on positivity | 600 µg/kg q24h 3d | 5.4 | 15.6 | 29.0 | 12.3 | -22.3 |
| IVM 600 | on peak | 600 µg/kg q24h 3d | 5.4 | 14.0 | 28.4 | 10.2 | -13.2 |
| LPV/r 400/100 | on positivity | 400/100 mg q12h 14d | 5.4 | 13.5 | 28.4 | 10.2 | -0.4 |
| LPV/r 400/100 | on peak | 400/100 mg q12h 14d | 5.4 | 13.5 | 28.4 | 10.2 | 0.0 |
| NTZ 1200 | on positivity | 1200 mg q6h 5d | 5.4 | 13.5 | 28.4 | 10.2 | 0.0 |
| NTZ 1200 | on peak | 1200 mg q6h 5d | 5.4 | 13.5 | 28.4 | 10.2 | 0.0 |
| NTZ 2900 | on positivity | 2900 mg q12h 5d | 5.4 | 13.5 | 28.4 | 10.2 | 0.0 |
| NTZ 2900 | on peak | 2900 mg q12h 5d | 5.4 | 13.5 | 28.4 | 10.2 | 0.0 |
| No treatment | NA | - | 5.4 | 13.5 | 28.4 | 10.2 | 0.0 |

# Supplementary Figures


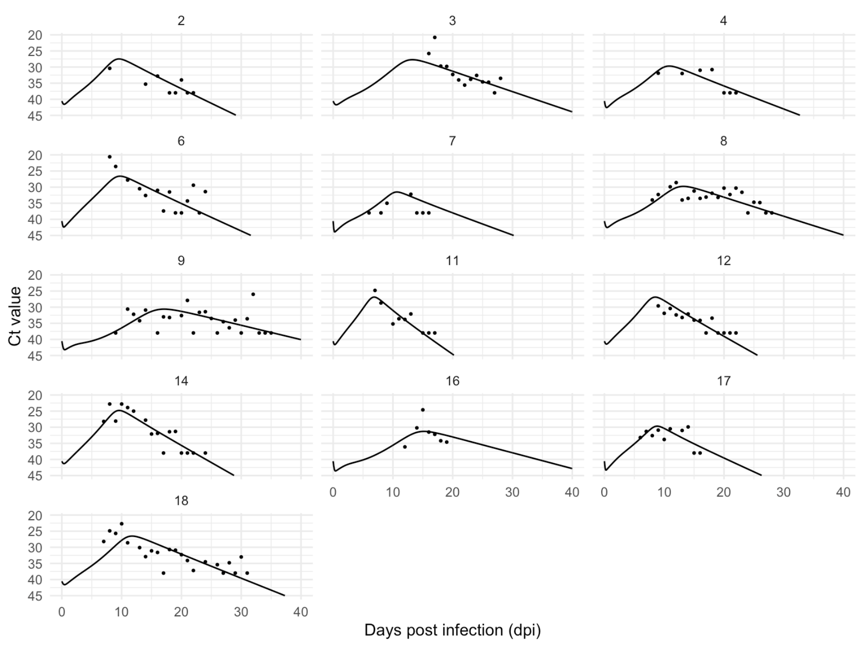


**Supplementary Figure S1** - Individual plots of serial cycle threshold (Ct) values by days post infection in SARS-CoV-2 of patients included in the analysis (n=13, dots) and model fit (line).


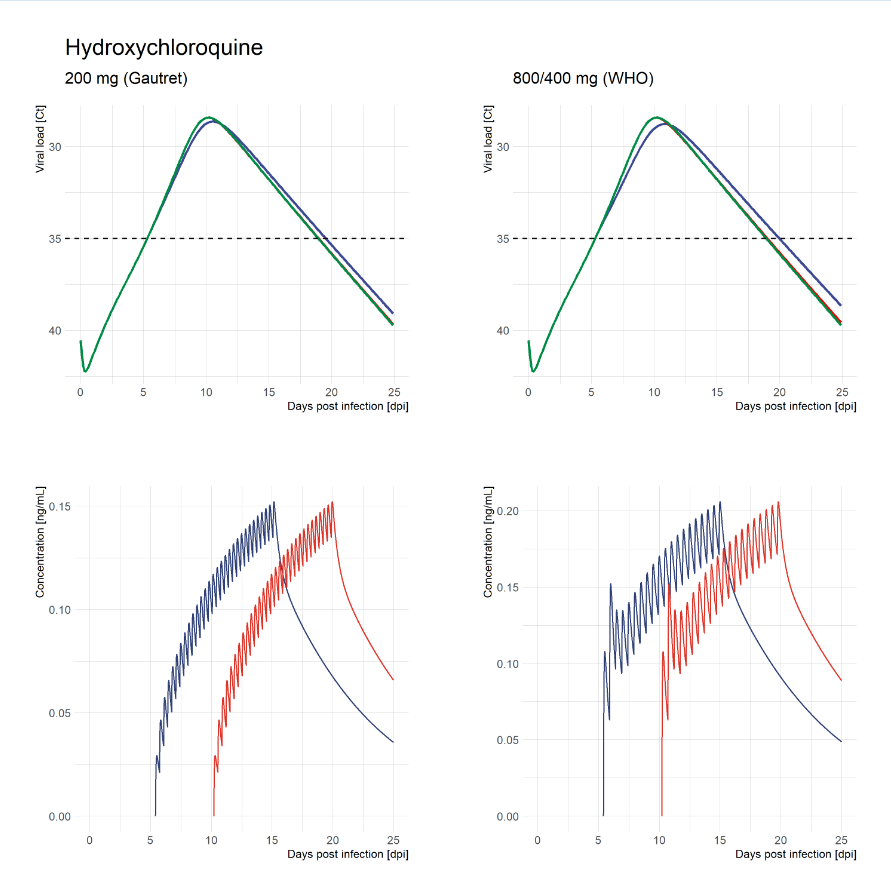


**Supplementary Figure S2** – Effect of Hydroxychloroquine pharmacokinetic on SARS-CoV-2 viral kinetics (blue: treatment on positivity, red: treatment on peak, green: untreated)


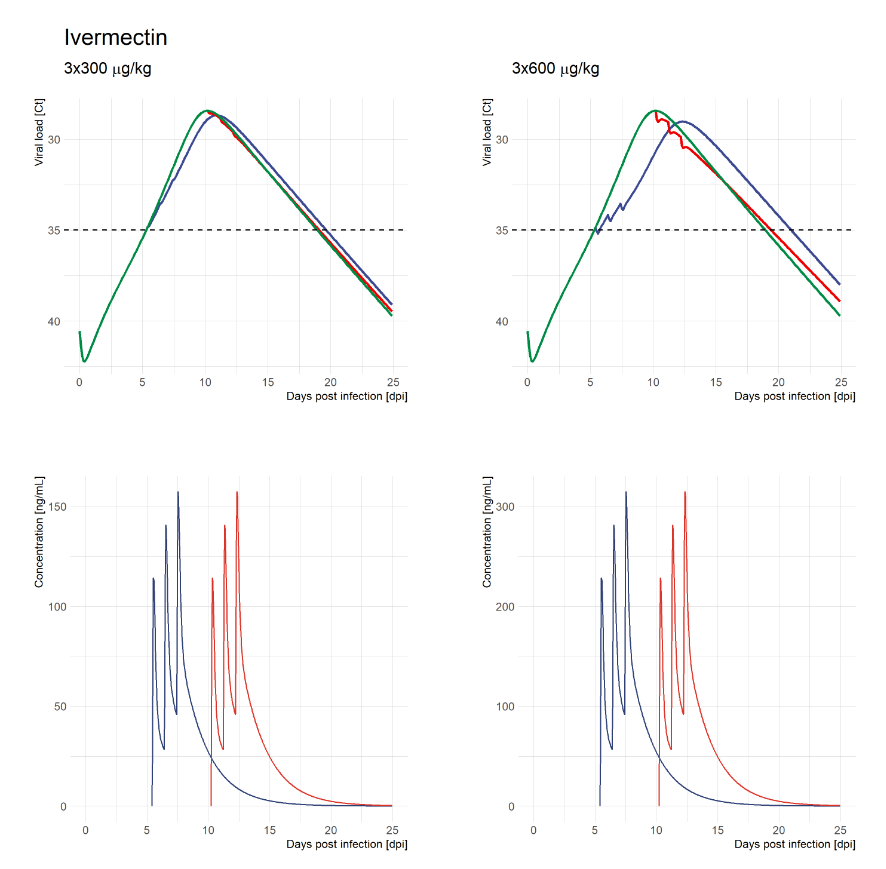


**Supplementary Figure S3** – Effect of ivermectin pharmacokinetic on SARS-CoV-2 viral kinetics (blue: treatment on positivity, red: treatment on peak, green: untreated)


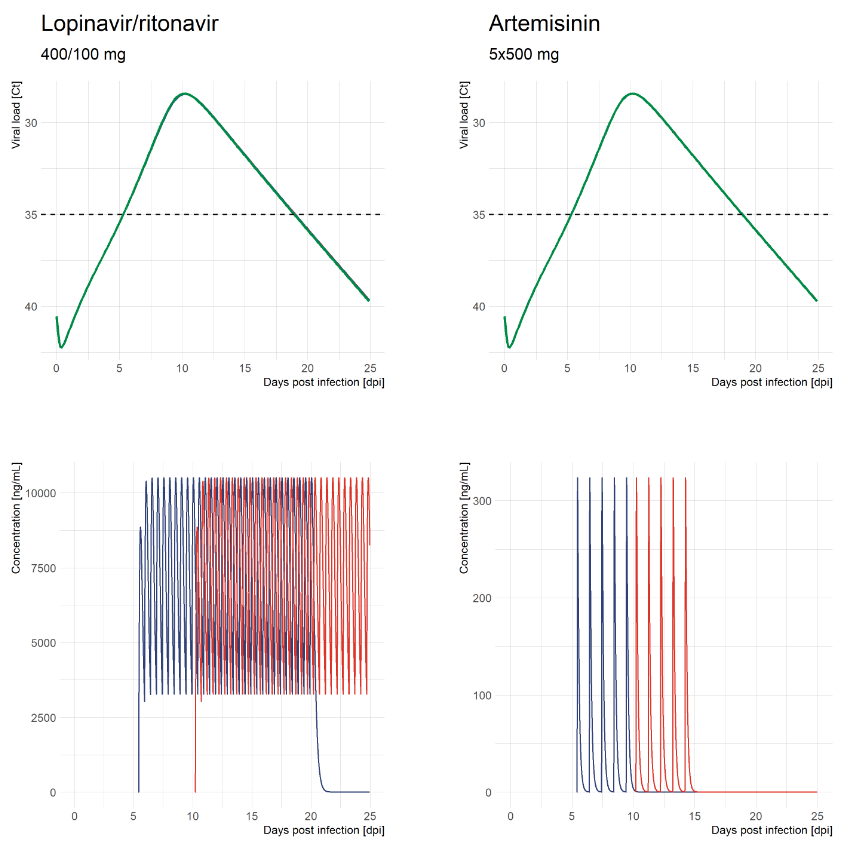


**Supplementary Figure S4** – Effect of lopinavir/ritonavir and Artemisinin pharmacokinetic on SARS-CoV-2 viral kinetics (blue: treatment on positivity, red: treatment on peak, green: untreated)


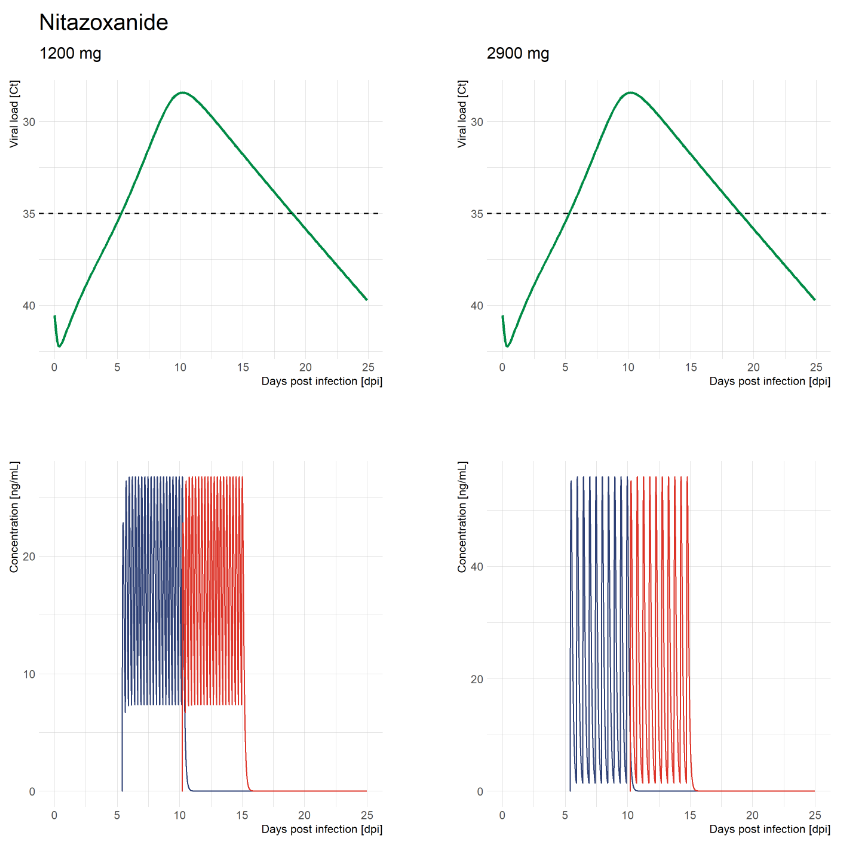


**Supplementary Figure S5** – Effect of nitazoxanide pharmacokinetic on SARS-CoV-2 viral kinetics (blue: treatment on positivity, red: treatment on peak, green: untreated)

# References

Atzori, C., Villani, P., Regazzi, M., Maruzzi, M., and Cargnel, A. (2003). Detection of intrapulmonary concentration of lopinavir in an HIV-infected patient. *AIDS (London, England)* 17**,** 1710-1711.

Boffito, M., Hoggard, P.G., Lindup, W.E., Bonora, S., Sinicco, A., Khoo, S.H., Di Perri, G., and Back, D.J. (2004). Lopinavir protein binding in vivo through the 12-hour dosing interval. *Ther Drug Monit* 26**,** 35-39.

Choy, K.-T., Wong, A.Y.-L., Kaewpreedee, P., Sia, S.F., Chen, D., Hui, K.P.Y., Chu, D.K.W., Chan, M.C.W., Cheung, P.P.-H., Huang, X., Peiris, M., and Yen, H.-L. (2020). Remdesivir, lopinavir, emetine, and homoharringtonine inhibit SARS-CoV-2 replication in vitro. *Antiviral research* 178**,** 104786-104786.

Degani-Katzav, N., Klein, M., Har-Even, M., Gortler, R., Tobi, R., and Paas, Y. (2017). Trapping of ivermectin by a pentameric ligand-gated ion channel upon open-to-closed isomerization. *Scientific Reports* 7**,** 42481.

Fda (2005). "PRESCRIBING INFORMATION: Alinia® (nitazoxanide) Tablets (nitazoxanide) for Oral Suspension ".).

Furst, D.E. (1996). Pharmacokinetics of hydroxychloroquine and chloroquine during treatment of rheumatic diseases. *Lupus* 5 Suppl 1**,** S11-15.

Jagdev S. Sidhu, M.A. (1997). Single-Dose, Comparative Study of Venous, Capillary and Salivary Artemisinin Concentrations in Healthy, Male Adults. *The American Journal of Tropical Medicine and Hygiene* 56.

Klotz, U., Ogbuokiri, J.E., and Okonkwo, P.O. (1990). Ivermectin binds avidly to plasma proteins. *Eur J Clin Pharmacol* 39**,** 607-608.

Li, C., Xu, J., Liu, J., and Zhou, Y. (2020). The within-host viral kinetics of SARS-CoV-2. *MBE* 17**,** 2853–2861.

Lifschitz, A., Virkel, G., Sallovitz, J., Sutra, J.F., Galtier, P., Alvinerie, M., and Lanusse, C. (2000). Comparative distribution of ivermectin and doramectin to parasite location tissues in cattle. *Veterinary Parasitology* 87**,** 327-338.

Liu, J., Cao, R., Xu, M., Wang, X., Zhang, H., Hu, H., Li, Y., Hu, Z., Zhong, W., and Wang, M. (2020). Hydroxychloroquine, a less toxic derivative of chloroquine, is effective in inhibiting SARS-CoV-2 infection in vitro. *Cell Discovery* 6**,** 16.

Long, Q.-X., Liu, B.-Z., Deng, H.-J., Wu, G.-C., Deng, K., Chen, Y.-K., Liao, P., Qiu, J.-F., Lin, Y., Cai, X.-F., Wang, D.-Q., Hu, Y., Ren, J.-H., Tang, N., Xu, Y.-Y., Yu, L.-H., Mo, Z., Gong, F., Zhang, X.-L., Tian, W.-G., Hu, L., Zhang, X.-X., Xiang, J.-L., Du, H.-X., Liu, H.-W., Lang, C.-H., Luo, X.-H., Wu, S.-B., Cui, X.-P., Zhou, Z., Zhu, M.-M., Wang, J., Xue, C.-J., Li, X.-F., Wang, L., Li, Z.-J., Wang, K., Niu, C.-C., Yang, Q.-J., Tang, X.-J., Zhang, Y., Liu, X.-M., Li, J.-J., Zhang, D.-C., Zhang, F., Liu, P., Yuan, J., Li, Q., Hu, J.-L., Chen, J., and Huang, A.-L. (2020). Antibody responses to SARS-CoV-2 in patients with COVID-19. *Nature Medicine* 26**,** 845-848.

Nair, M.S., Huang, Y., Fidock, D.A., Polyak, S.J., Wagoner, J., Towler, M.J., and Weathers, P.J. (2021). *Artemisia annua* L. extracts prevent *in vitro* replication of SARS-CoV-2. *bioRxiv***,** 2021.2001.2008.425825.

Rajoli, R.K., Pertinez, H., Arshad, U., Box, H., Tatham, L., Curley, P., Neary, M., Sharp, J., Liptrott, N.J., Valentijn, A., David, C., Rannard, S.P., Aljayyoussi, G., Pennington, S.H., Hill, A., Boffito, M., Ward, S.A., Khoo, S.H., Bray, P.G., O'neill, P.M., Hong, W.D., Biagini, G., and Owen, A. (2020). Dose prediction for repurposing nitazoxanide in SARS-CoV-2 treatment or chemoprophylaxis. *medRxiv : the preprint server for health sciences***,** 2020.2005.2001.20087130.

Wang, M., Cao, R., Zhang, L., Yang, X., Liu, J., Xu, M., Shi, Z., Hu, Z., Zhong, W., and Xiao, G. (2020). Remdesivir and chloroquine effectively inhibit the recently emerged novel coronavirus (2019-nCoV) in vitro. *Cell Research* 30**,** 269-271.
